# The quality of guidelines for diabetic foot ulcers: A critical appraisal using the AGREE II instrument

**DOI:** 10.1371/journal.pone.0217555

**Published:** 2019-09-23

**Authors:** Peiying Zhang, Qian Lu, Huijuan Li, Wei Wang, Gaoqiang Li, Longmei Si, Yanming Ding

**Affiliations:** 1 Nursing Department, Peking University First Hospital, Beijing, China; 2 School of Nursing, Peking University, Beijing, China; 3 Department of Plastic and Burns, Peking University First Hospital, Beijing, China; 4 Schhol of Medicine, Tongji University, Shanghai, China; Universitat Witten/Herdecke, GERMANY

## Abstract

This study aims to evaluate the quality of clinical practice guidelines(CPGs) for patients with diabetic foot worldwide. A search of guidelines websites, databases and academic institutions websites was performed from January 1^st^, 2010, until June 30^th^, 2018. Four assessors independently rated the quality of each CPG using the Appraisal of Guidelines for Research and Evaluation (AGREE) II instrument. Twelve CPGs satisfied the inclusion criteria. The median scores for the 6 AGREE II domains (scope and purpose, stakeholder involvement, rigor of development, clarity of presentation, applicability, and editorial independence) were 92.5%, 72.5%, 71.5%, 89%, 47%, and 77%, respectively. The overall quality of the CPGs was good since the majority of the CPGs reached an overall guideline quality between 5 and 7 points. Different CPGs had widely varying scores in the same area, ranging from 25 to 94 points.

## Introduction

Diabetic foot (DF) is a common and serious complication of diabetes mellitus. The prevalence of diabetic foot ulceration is about 6.3% worldwide[1]. Individuals with diabetes and DF have been reported to be three times more likely to die at any time than those with diabetes who do not have DF [2]. Such patients undergo suffering and bear an enormous economic burden [3, 4]. Standardized and scientific treatment can improve patient outcomes, save medical resources, and reduce unnecessary costs for patients. However, there is great variation in the treatment and management of DF in different areas and hospitals. High-quality CPGs for use in clinical practice are recommended as a decision-making tool. In 1990, the Institute of Medicine (IOM) provided a definition for clinical practice guidelines, which were systematically developed statements to assist practitioner and patient decisions regarding appropriate health care for specific clinical circumstances [5]. As the best methods for guideline development have evolved, the IOM updated the definition of clinical practice guidelines which states that “clinical practice guidelines are statements that include recommendations intended to optimize patient care that are informed by a systematic review of evidence and an assessment of the benefits and harms of alternative care options” [6]. This new definition better reflects the current consensus on what constitutes a clinical practice guideline. A high-quality CPG must be based on systematic review and balance benefits and drawbacks. However, the non-systematic creation of guidelines can lead to considerable variation, with implications for the quality of care and clinical decision-making [7]. Therefore, finding CPGs regarding DF and evaluating their quality are essential. CPGs help reduce inappropriate practice variation, promote the translation of research into practice, and improve health care quality and safety[6].

The Appraisal of Guidelines for Research and Evaluation (AGREE) is the instrument designed to assess the quality of the process and reporting of CPG development, and was released in 2003 [8]. The AGREE instrument has been translated into many languages and was cited in more than 100 publications until 2009[9]. Later, to strengthen the measurement properties of AGREE and to better meet the needs of the intended users, the AGREE II was developed [9]. This instrument has been widely used to appraise CPGs worldwide [10–12]. In 2013, the AGREE II was translated into Chinese[13]. We performed a search for DF-related CPGs and assessed their quality using the Chinese version of the AGREE II instrument. The objective of the AGREE II assessment is to clarify the methodological quality of the DF CPGs in order to help health professional decide on the selection of good CPGs.

## Materials and methods

### Search strategy

We performed a systematic search of major websites, electronic databases, and academic institutions that published guidelines for CPGs from January 1^st^,2010, until June 30^th^, 2018. These databases included the National Guideline Clearinghouse (NGC), Registered Nurses’ Association of Ontario (RNAO), National Institute for Health and Clinical Excellence (NICE), Scottish Intercollegiate Guidelines Network (SIGN), New Zealand Guideline Group (NZGG), Guidelines International Network (GIN), Centers for Disease Control and Prevention (CDC), World Council of Enterostomal Therapists (WCET), Wound, Ostomy, and Continence Nurses Society (WOCN), Wound Healing Society (WHS), National Health and Family Planning Commission of the People’s Republic of China, PubMed, ProQuest, Web of Science, Cumulative Index to Nursing and Allied Health Literature (CINAHL), Best Practice, China National Knowledge Infrastructure (CNKI), VIP China Science and Technology Journal Database, Wanfang Database, and Medlive.

We used the following Medical Subject Headings and free terms as the English search terms: ((‘foot disease’ or ‘foot ulcer’ or ‘diabetic foot’ or ‘foot infections’ or ‘foot problems’ or ‘foot complications’) or ((foot and ‘diabetes mellitus’) or diabetes or diabetic) and guideline). The Chinese search terms were (‘diabetic foot’ and guideline).

Take the searching formula used for PubMed as an example:

#1 “diabetes mellitus”[Mesh]
#2 diabetes or diabetic[TI/AB]
#3 “foot” or “foot disease” or “foot ulcer” or “foot problem” or “foot complication”[TI/AB]
#4 “diabetic foot”[Mesh]
#5 ((#1 or #2) and (#3 or #4)) and guideline?[TI/AB]

### Inclusion and exclusion criteria

The inclusion criteria were as follows: (1) clinical practice guidelines(guidelines developed based on systematic reviews of the literatures, assessing the scientific quality of the available evidence, and rating the strength or weakness of the final recommendation)[14], (2) guidelines in the Chinese and English language, (3)CPGs that gave mainly recommendations for DF, and (4) if the CPG was updated, only the most recent version was included in the study.

CPGs were excluded if they were incomplete guidelines (e.g., parts of a CPG), translations of guidelines in other languages, duplicate publications, or summaries of several guidelines.

### Appraisal of guidelines

The AGREE II instrument is used to assess the methodological rigor and transparency of CPGs. It consists of 23 items grouped into the following six domains and two overall assessment items. The six domains are scope and purpose, stakeholder involvement, rigor of development, clarity of presentation, applicability, and editorial independence. The overall assessment includes a rating of the overall quality of the guideline and whether the guideline would be recommended for use in practice [15]. We also made an effort to find the CPGs’ methodological manuals related to the included CPGs, as suggested by the AGREE II Group (The AGREE Next Step Consortium, 2009). These supporting materials are sometimes contained in the same document as the guideline recommendations or it may be summarized in a separate report. Therefore, we downloaded theses materials where the CPGs suggested. Each of the AGREE II items and the global rating item are rated on a numeric scale between 1 for strongly disagree to 7 for strongly agree. Scores of 2 and 6 can be assigned if the item does not meet the full criteria or considerations. Assessors are suggested to complete each item. The scores for the six domains are independent and should not be added together to assess CPG quality. Each domain score is a standardized score calculated as follows: scaled domain score = (obtained score–minimum possible score) / (maximum possible score–minimum possible score) × 100%. The AGREE consortium has not set a criterion to differentiate high-quality CPGs from poor-quality CPGs. Decisions must be made by the users and should be guided by the context of the CPG. A score of 5–7 points was defined as good quality, a score of 3–4 points was defined as fair quality, and a score of 1–2 points was defined as poor quality. The user is also asked to give an overall score of the CPG and whether he/she would recommend using the guideline [9].

The CPGs were evaluated independently by four reviewers. The four Chinese assessors received education regarding the guideline development process and evidence-based nursing and were trained on the use of AGREE II. To ensure that each assessor understood each item, a pilot test that consisted of an appraisal of one of the DF CPGs was administered. After the evaluation, answers of four people were compared, the score difference for each item greater than 2 points or someone gave score of 1 and the other reviewer(s) score of 2 or more on the same item was defined as a large difference. Then the four reviewers were asked to find out the supporting information on CPGs or their attachments on the items of large differences. Next, four reviewers were asked to give a new score after discussion. If the new scores still did not meet the requirements, we would ask a professor with had rich experience in using AGREEIIfor help. The fifth reviewer would combine all the supporting materials and opinions of the four reviewers to give a final score. Reliability of the scores assigned by the four reviewers was evaluated using intra-class correlation coefficients (ICCs) with a 95% confidence interval (CI). Statistical analysis was performed using SPSS version 20.0 (IBM Corp., Armonk, NY, USA).

## Results

**Fig 1:**
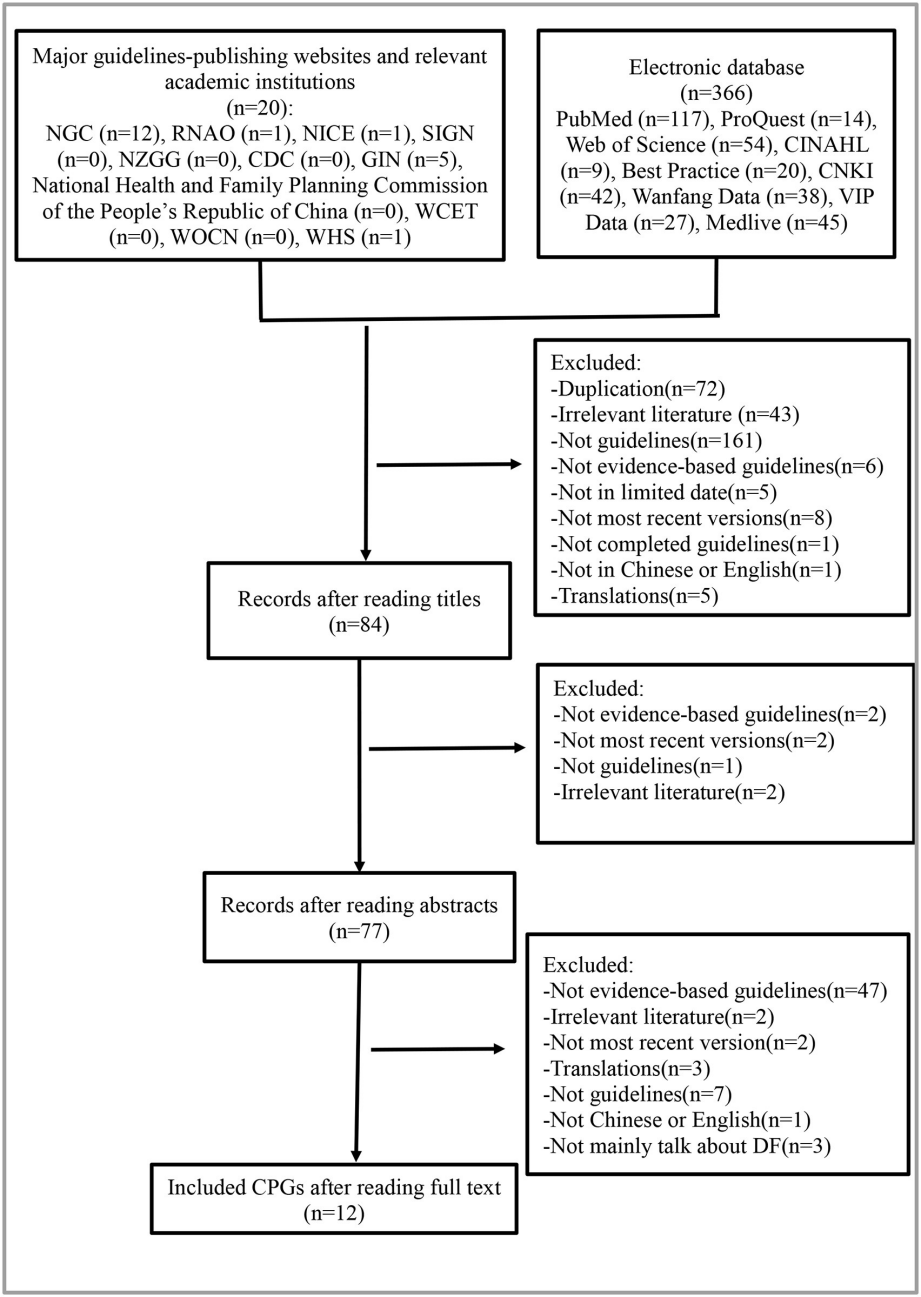
PRISMA diagram for the included studies.

Twelve CPGs meeting the criteria were included in our study. Five CPGs issued by the International Working Group on the Diabetic Foot (IWGDF) covered five aspects of DF. These CPGs were developed using the same method. They were therefore evaluated as one CPG. A flowchart illustrating our search of the CPGs is shown in Fig 1.

### Guideline characteristics

America was the country of origin for four sets of CPGs[16–19], WHS’s CPGs focued on diagnosis, management of diabetic foot including offloading, infection, wound treatment and use of adjuvant agents for diabetic foot treatment. The UHMS CPGs focused on hyperbaric oxygen therapy for the treatment of diabetic foot and the IDSA CPGs mainly provide recommendations for diagnosis, assessment, and treatment of patients with diabetic foot. One’s country of origin was Great Britain [20], recommendations regarding the prevention, infection, wound treatment, Charcot arthropathy were given; one’s country of origin was Canada [21], these recommendations focused mainly on the assessment and management of history of disease, wound treatment, vascular disease, peripheral neuropathy, and offloading. This CPG also emphasized on the clinical implementation process, and also a user toolkit. One’s country of origin was Japan [22], it mainly covered the treatment of infections, management of osteomyelitis, peripheral neuropathy, peripheral arterial disease, wound management and information on offloading. And five CPGs were from international academic societies [23–27], and these five CPGs were targeted at five aspects of diabetic foot, including prevention, offloading, infection, peripheral arterial disease, and wound management. Almost all of the CPGs used the Grading of Recommendations Assessment, Development and Evaluation system, while three guidelines used their own grading standards. All of the CPGs were developed by multidisciplinary teams or societies. General information regarding the CPGs is listed in Table 1.

**Table 1:** General information regarding the CPGs.

Number	Countries	Guidelines	Releasing Institution	Year of publication or update
1	America	The management of diabetic foot: A clinical practice guideline by the Society for Vascular Surgery in collaboration with the American Podiatric Medical Association and the Society for Vascular Medicine (Hingorani et al., 2016)	APMA^a^, SVS^b^, and SVM^c^	2016
2	America	WHS Guidelines Update: Diabetic Foot Ulcer Treatment Guidelines (Lavery et al., 2016)	WHS^d^	2016
3	International	IWGDF guidance on the prevention of foot ulcers in at-risk patients with diabetes (Bus, van Netten, et al., 2016)	IWGDF^e^	2015
4	International	IWGDF guidance on footwear and offloading interventions to prevent and heal foot ulcers in patients with diabetes (Bus, Armstrong, et al., 2016)	IWGDF	2015
5	International	IWGDF guidance on the diagnosis and management of foot infections in persons with diabetes (Lipsky et al., 2016)	IWGDF	2015
6	International	IWGDF guidance on the diagnosis, prognosis and management of peripheral artery disease in patients with foot ulcers in diabetes (Hinchliffe et al., 2016)	IWGDF	2015
7	International	IWGDF guidance on use of interventions to enhance the healing of chronic ulcers of the foot in diabetes (Game et al., 2016)	IWGDF	2015
8	Britain	Diabetic foot problems: prevention and management (National Institute for Health and Care Excellence, 2015)	NICE^f^	2015
9	America	A clinical practice guideline for the use of hyperbaric oxygen therapy in the treatment of diabetic foot ulcers (Huang et al., 2015)	UHMS^g^	2015
10	Canada	Assessment and management of foot ulcers for people with Diabetes second edition (Registered Nurses’ Association of Ontario, 2013)	RNAO^h^	2013
11	Japan	The wound/burn guidelines-3: Guidelines for the diagnosis and treatment for diabetic ulcer/gangrene (Isei et al., 2016)	JDA^i^	2012
12	America	2012 Infectious Diseases Society of America Clinical Practice Guideline for the Diagnosis and Treatment of Diabetic Foot Infections (Lipsky et al., 2012)	IDSA^j^	2012

^a^APMA, American Podiatric Medical Association.

^b^SVS, Society for Vascular Surgery.

^c^SVM, Society for Vascular Medicine.

^d^WHS, wound healing society.

^e^IWGDF, International Working Group on the Diabetic Foot.

^f^NICE, National Institute for Health and Clinical Excellence.

^g^UHMS, Undersea and Hyperbaric Medical Society.

^h^RNOA, Registered Nurses’ Association of Ontario.

^i^JDA, Japanese Dermatology Association.

^j^IDSA, Infectious Diseases Society of America.

### Appraisal of the AGREE II domains of the guidelines

The results of the evaluation of the CPGs using the AGREE II instrument are shown in Table 2. The ICC of the four reviewers are reported in Table 3. All of the ICC values are greater than 0.75, indicating good reliability among the four reviewers [28].

**Table 2:** Scaled domain percentages in the AGREE II for the different CPGs.

CPG-Number	Scores, %						Overall assessment	Recommendation for use
	Scope and purpose	Stakeholder involvement	Rigor of development	Clarity and presentation	Applicability	Editorial independence		
1	97	81	80	94	46	33	5	Yes
2	75	46	53	72	27	2	4	Yes(With Modifications)
3–7	96	85	78	97	75	96	7	Yes
8	99	89	58	85	74	96	6	Yes
9	92	97	77	92	40	52	5	Yes
10	93	64	88	86	86	81	7	Yes
11	72	28	49	82	31	73	4	Yes(With Modifications)
12	90	60	66	92	48	94	6	Yes
Median (Range)	92.5 (27)	72.5 (69)	71.5 (39)	89 (25)	47 (59)	77 (94)		

**Table 3:** Inter-class reliability of each guideline.

CPG-Number	ICC (95% CI)	F value	P value
1	0.765 (0.600–0.882)	17.202	<0.001
2	0.756 (0.595–0.875)	15.433	<0.001
3–7	0.754 (0.589–0.874)	15.75	<0.001
8	0.763 (0.594–0.881)	17.325	<0.001
9	0.776 (0.626–0.885)	16.821	<0.001
10	0.757 (0.605–0.874)	14.556	<0.001
11	0.791 (0.646–0.894)	18.613	<0.001
12	0.785 (0.597–0.897)	22.180	<0.001

The overall quality of the CPGs is good for the majority of the CPGs reach an overall quality between 5 and 7 points. The five CPGs by IWGDF and that by RNAO have scored greater than 60% in all six domains [21, 23–27]. The JDA and WHS CPGs had domains which scored less than 30% in applicability, editorial independence, and stakeholder involvement [18, 22]. Others receive scores between 30% and 60% in six domains. The twelve CPGs had median scores of 92.5%, 72.5%, 71.5%, 89%, 47%, and 77% in the six AGREE II domains (scope and purpose, stakeholder involvement, rigor of development, clarity of presentation, applicability, and editorial independence). Different CPGs had widely varying scores in the same area, ranging from 25% to 94%. In all CPGs, the domain “Scope and purpose” received the highest score of 92.5% and the domain “Applicability” received the lowest score of 47%. However, we were unable to find the manuals for two of the CPGs [18, 22].

### Scope and purpose

According to the AGREE II instrument, this domain evaluated whether the overall objectives, health questions, and target population of the guidelines are described specifically. Ten of the CPGs had scored greater than 90% and two had scores greater than 70%. The two guidelines were those without methodological manuals [18, 22].

### Stakeholder involvement

This domain assesses whether all relevant professional groups are involved in the guideline development group, whether the guidelines consider the views of the target population, and whether the guidelines define the target users clearly. The CPG by the Undersea and Hyperbaric Medical Society (UHMS) received a nearly full score of 97% in this domain. Eight of the guidelines had scores greater than 60%[16, 20, 21, 23–27]. A common problem is that the roles of the experts in the development of the guidelines are not clarified [16, 18, 19, 22–27]. Only the guideline by the UHMS provided details regarding how the target population’s views were assessed. The users were identified clearly in most of the guidelines.

### Rigor of development

This domain is used to assess whether the guideline formation process follows a rigorous methodology including a systematic search strategy, criteria for selecting evidence, strengths and limitations of the body of evidence, methods for formulating recommendations, benefits and harms considerations, provision of an explicit link between the recommendations and evidence, external review, and updating procedures. Three of the CPGs lacked full search strategies. This was why we suggested that the guidelines should include an attachment containing a full search strategy [16, 18, 22]. Three of the CPGs had unclear reports of the criteria used to select evidence [18, 19, 22]. Most of the CPGs, with the exception of those by the IWGDF and UHMS, did not include paragraphs or chapters describing the risks and biases of studies. Almost all of the guidelines include only a small amount of information regarding the recommendation development process. Three of the CPGs describe an explicit link between evidence and recommendation, such as a table of the body of evidence or evidence summaries [16, 17, 27]. One CPG [17] provided details regarding the external review collection process, while the others did not provide a specific description of the external review process or only mentioned external review without providing a specific description. Three of the guidelines include a procedure for updating[20–22]. Overall, nine of the CPGs receive scores greater than 60% in this domain.

### Clarity of presentation

This domain is used to determine whether the descriptions of the recommendations are clear, specific, and easily identifiable. All of the CPGs scored high in this domain, where the median score is 89%.

### Applicability

This domain emphasizes the guidelines’ application using criteria such as “facilitators and barriers”, “advice or tools can be put into practice”, “potential resource in application”, and “monitor or auditing criteria”. The median score for this domain was 47%. RNAO has developed a toolkit[29] to support the systematic implementation of CPGs containing facilitator and barrier analysis as well as tools such as PUSH tool or wound assessment form in it that could be put into practice. The IWGDF had developed a set of training courses for patients[30] with DF and health-care providers to facilitate guideline implementation, but it ignored facilitators and barriers. Others do not perform well in this domain. Overall, most of the guidelines, with the exception of the guideline by the RNAO, lacked applicability, which can limit implementation of the guidelines.

### Editorial independence

The information regarding “the funding body” and “competing interest of guideline development group” in the guidelines and/or in the supporting documents are appraised by AGREEII. are assessed in this domain. Half of the CPGs reported their sponsors and include statements explaining that the sponsors did not influence the recommendations. Most of the guidelines did not include sufficient information regarding the methods by which potential competing interests were evaluated. However, the guidelines by NICE and IWGDF performed very well in this domain.

## Discussion

This study aims to evaluate the quality of clinical practice guidelines for diabetic foot worldwide. We identified twelve CPGs that discuss DF developed based on evidence from 2012 until now. The median scores for the 6 AGREE II domains (scope and purpose, stakeholder involvement, rigor of development, clarity of presentation, applicability, and editorial independence) were 92.5%, 72.5%, 71.5%, 89%, 47%, and 77%, respectively. The overall quality of the CPGs was good for the majority of the CPGs reached an overall guideline quality between 5 and 7 points. Different CPGs had widely varying scores in the same area, ranging from 25% to 94%. Clinical guidelines could be effective in improving the care provided to patients [31, 32]. The CPGs for DF performed well in the domains of “scope and purpose” and “clarity of guideline”, as also reported in other studies[12, 33]. Development of CPGs must be performed by a team, and potential participants in the development of the guideline should include clinicians, content experts, researchers, and policymakers. Most commonly, guideline development groups consist of 10 to 20 members from 3 to 5 relevant disciplines [6]. A rigorous and transparent process for the development of guidelines is essential [34, 35]. The CPGs here had moderate rigor scores, as the median score in this domain was 71.5%. Systematic search for evidence, explicit inclusion and exclusion criteria, and the grading of evidence are basic components of rigor in guideline development. However, half of the CPGs lost points for having little information regarding these components. CPGs are expected to report specific methodological information regarding external review by experts and on how to formulate recommendations. The best way to interpret the link between the recommendations and the supporting evidence is to present evidence summaries or tables to the users in the guideline. A common problem presented in the CPGs studied here is that they do not contain sufficient explanations regarding the application of guidelines. This was an issue that was also regularly observed in other fields of research [10, 12, 36]. In the applicability domain, facilitating the efficient use of these evidence-based resources is also a key point during the development of CPGs. Future CPGs are expected to identify barriers and facilitators in implementing guidelines, provide tools or other resources promoting implementation, and provide cost information on the recommendations. The CPG from the RNAO set a good example in this respect. Of course, some conceptual models can also guide the implementation of CPGs for researchers and clinical staff. These models include the Knowledge-to-Action model [33], Ottawa model [37], and integrated–Promoting Action on Research Implementation in Health Services model [38]. The process of obtaining information regarding conflict of interest and its description should also be strengthened. We suggest that there should be a scientific development standard for guideline developers. The AGREE II may also be used as a guide for guideline development. Finally, we suggest that all developers upload attachments when issuing the guidelines. This would enhance the quality of the methodology and transparency.

### Limitations

The CPGs that satisfied our inclusion criteria were assessed using the AGREE II instrument. Although the quality of methodology of a guideline is not equal to its content quality, the AGREE II instrument is a validated and international widely used instrument for guideline assessment currently. This paper is solely a quality assessment of CPGs and the quality of the recommendations listed within the CPGs has not been assessed. Further, it should be noted that high quality CPGs including appropriate methodologies in their development may not give necessarily guarantee for high quality or appropriate recommendations(content quality).

## Conclusions

The CPGs for DF demonstrated good quality, although their applicability and reporting quality should be strengthened. In all guidelines, the domain “Scope and purpose” receives the highest score of 92.5% and the domain “Applicability” receives the lowest score of 47%. With this appraisal of current clinical practice guidelines for DF, Health care professionals can not only recognize DF CPGs, but also know which CPGs are more trustworthy and of potential high quality.

## Supporting information

S1 Attachment The search strategies in each database and the inclusion and exclusion of articles. (DOCX)

S1 Dataset The included articles in each databasse. (RAR)
