# Supplementary material for: The quality of guidelines for diabetic foot ulcers: A critical appraisal using the AGREE II instrument
Source: PLoS One. 2019 Sep 23;14(9):e0217555. doi: 10.1371/journal.pone.0217555 (PMC6756510; doi:10.1371/journal.pone.0217555)

1. NGC(n=12):

The NGC did not work because of the fund for it has been ended by US. So the there is no new retrieval in NGC.

Key words: “foot”

Excluded(n=2): for this literature is not for diabetic foot.

for this literature is not inlimited date.

Included(n=10):

1. RNAO(n=1)：

Key words: “foot”

Included(n=1)： Assessment and management of foot ulcers for people with diabetes, second edition.

1. NICE(n=1):

Key words: “foot”

Included: Diabetic foot problems: prevention and management

1. GIN(n=5):

Key words: “diabetic foot”

| Titles | Year | Reason for included or excluded. |
| --- | --- | --- |
| [Reducing Foot Complications for People with Diabetes](https://www.g-i-n.net/library/international-guidelines-library/guidelines/rnao-ca/reducing-foot-complications-for-people-with-diabetes) | 2004 | Excluded. Not in research date. |
| National klinisk retningslinje for udredning og behandling af diabetiske fodsår | 2013 | Excluded.  Not in English or Chinese. |
| Management of Diabetic Foot | 2004 | Excluded.  Not in research date. |
| Diagnosis and Treatment of Diabetic Foot Infections | 2012 | Included |
| Diabetic foot problems: prevention and management | 2015 | Included |

Flow chart:


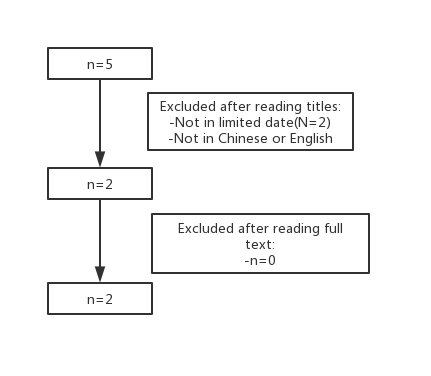


1. WHS(n=1)

Searched in its guideline library.

Included: WHS Guidelines update：diabetic foot ulcer treatment guidelines

1. PubMed(n=117):

Search strategy:

**(((((((foot[Title/Abstract] OR "foot disease"[Title/Abstract] OR "foot ulcer"[Title/Abstract] OR "foot problem"[Title/Abstract] OR "foot complication"[Title/Abstract])) OR diabetic foot[MeSH Terms])) AND (((foot[Title/Abstract] OR "foot disease"[Title/Abstract] OR "foot ulcer"[Title/Abstract] OR "foot problem"[Title/Abstract] OR "foot complication"[Title/Abstract])) OR diabetic foot[MeSH Terms])) AND guideline[Title/Abstract])) AND ("2010/1/1"[Date - Publication] : "2018/6/1"[Date - Publication])**

The data of results is as follows:

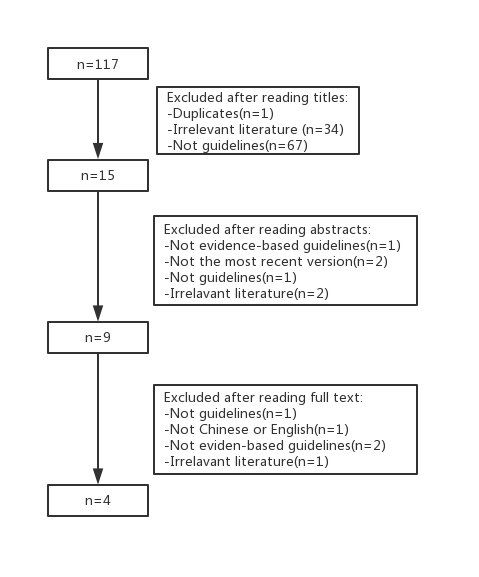


1. ProQuest(n=14)

Search strategy:

(((((((su(diabetes mellitus) OR noft(diabetes OR diabetic)) AND stype.exact("Magazines" OR "Trade Journals" OR "Scholarly Journals" OR "Dissertations & Theses")) AND la.exact("English" OR "Chinese")) AND stype.exact("Magazines" OR "Trade Journals" OR "Scholarly Journals" OR "Dissertations & Theses")) AND la.exact("English" OR "Chinese")) AND pd(20100101-20180630)) AND ((((((noft(foot OR "foot disease" OR "foot ulcer" OR "foot problem" OR "foot complication") OR mainsubject(diabetic foot)) AND stype.exact("Magazines" OR "Trade Journals" OR "Scholarly Journals" OR "Dissertations & Theses")) AND la.exact("English" OR "Chinese")) AND stype.exact("Magazines" OR "Trade Journals" OR "Scholarly Journals" OR "Dissertations & Theses")) AND la.exact("English" OR "Chinese")) AND pd(20100101-20180630)) AND (ti(guideline) AND stype.exact("Magazines" OR "Trade Journals" OR "Scholarly Journals" OR "Dissertations & Theses") AND la.exact("English" OR "Chinese"))) AND (at.exact("Instructional Material/Guideline") AND la.exact("ENG") AND pd(20100101-20180630))

The results website: https://search.proquest.com/search/1543816?accountid=13151

| Titles | Year | Excluded after reading titles | Excluded after reading full texts. |
| --- | --- | --- | --- |
| Diabetic Foot Australia guideline on footwear for people with diabetes. | 2018 |  | Excluded. Not evidence-based guidelines. |
| The management of diabetic foot: A clinical practice guideline by the Society for Vascular Surgery in collaboration with the American Podiatric Medical Association and the Society for Vascular Medicine | 2016 |  | Included. |
| The wound/burn guidelines - 3: Guidelines for the diagnosis and treatment for diabetic ulcer/gangrene. | 2016 |  | Included. |
| Specific guidelines for the diagnosis and treatment of peripheral arterial disease in a patient with diabetes and ulceration of the foot 2011 | 2012 |  | Excluded. Not evidence-based guidelines. |
| WHS guidelines update: Diabetic foot ulcer treatment guidelines | 2016 |  | Included. |
| 2012 Infectious Diseases Society of America clinical practice guideline for the diagnosis and treatment of diabetic foot infections. | 2012 |  | Included. |
| Practical guidelines on the management and prevention of the diabetic foot 2011 | 2012 |  | Excluded. Not evidence-based guidelines. |
| Specific guidelines for the treatment of diabetic foot infections 2011 | 2012 |  | Excluded. Not evidence-based guidelines. |
| What's new in the literature: an update of new research since the original WHS diabetic foot ulcer guidelines in 2006. | 2014 | Excluded. Not guidelines. |  |
| Guideline for the management of wounds in patients with lower-extremity neuropathic disease: an executive summary. | 2013 | Excluded. Not guidelines. |  |
| A clinical practice guideline for the use of hyperbaric oxygen therapy in the treatment of diabetic foot ulcers | 2015 |  | Included. |
| Specific guidelines on wound and wound-bed management 2011 | 2012 |  | Excluded. Not evidence-based guidelines. |
| British Association of Dermatologists' guidelines for the management of onychomycosis 2014. | 2014 | Excluded. Irrelevant literatures. |  |
| 3rd Guideline for Perioperative Cardiovascular Evaluation of the Brazilian Society of Cardiology | 2017 | Excluded. Irrelevant literatures. |  |


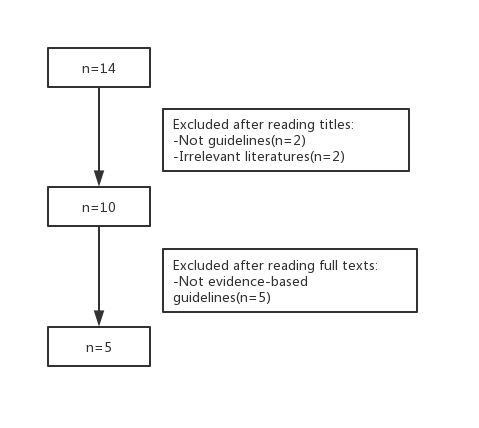


1. Web of Science (n=54):

Search strategy:

#1 TS=diabetes mellitus

#2 TS=diabetes or diabetic

#3 TS=foot or "foot disease" or "foot ulcer" "foot problem" or "foot complication"

#4 TS=diabetic foot

#5 (#1 or #2) and (#3 or #4) and TI=guideline


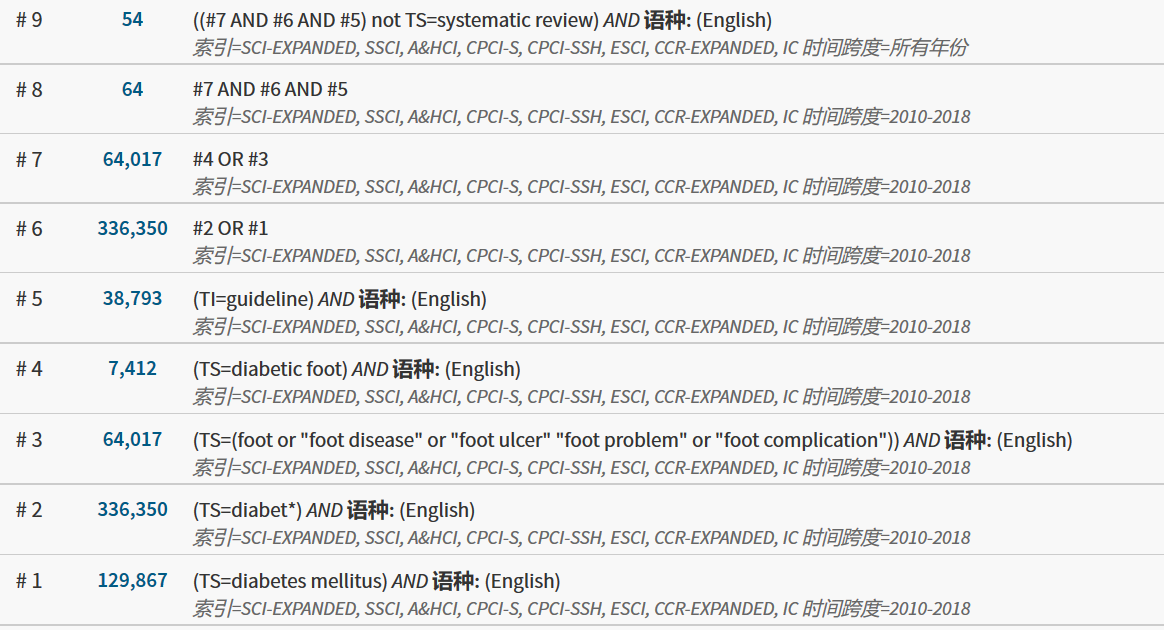


Original database:

Excluded after reading titles(n=41):

Excluded after reading abstracts(n=1):

Excluded after reading full texts(n=9):

Flow chart:


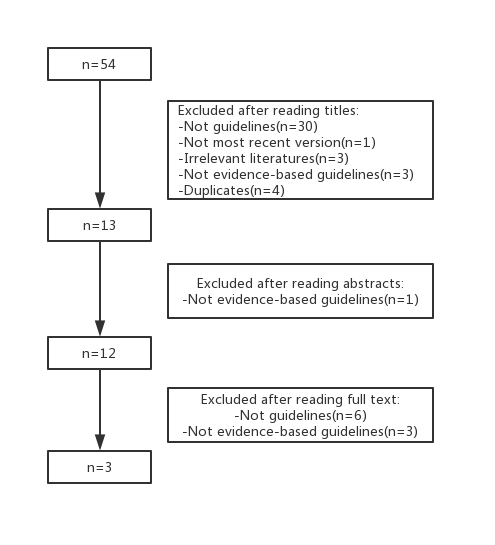


1. CINAHL(n=9)

Search strategy:


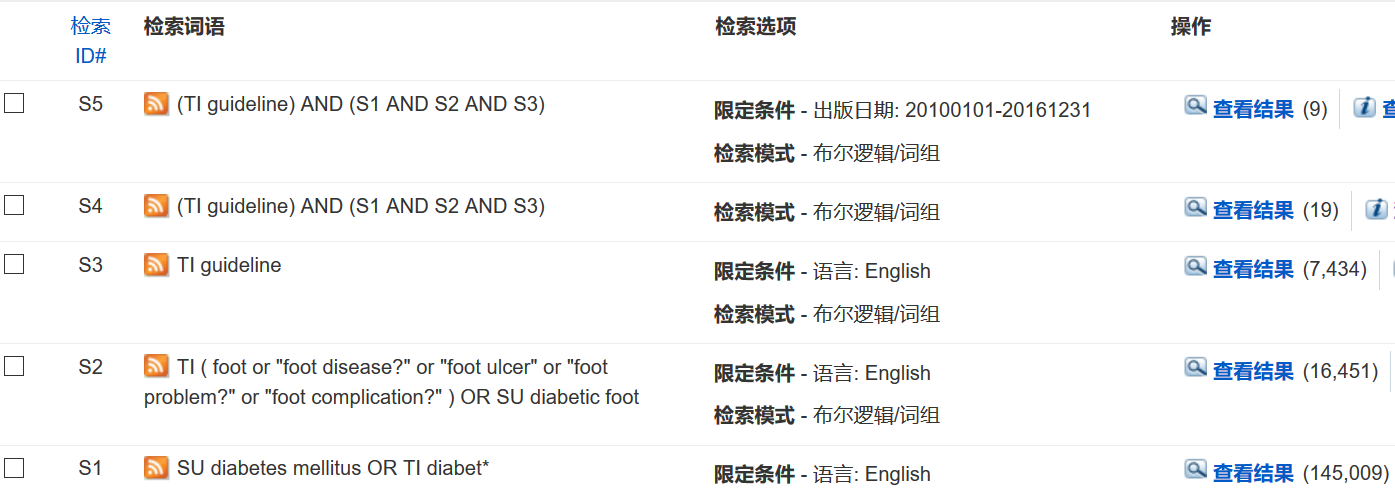


http://search.ebscohost.com/login.aspx?direct=true&db=rzh&bquery=(TI+guideline)+AND+(((SU+diabetes+mellitus)+OR+(TI+diabet*))+AND+((TI+(foot+OR+%26quot%3bfoot+disease%3f%26quot%3b+OR+%26quot%3bfoot+ulcer%26quot%3b+OR+%26quot%3bfoot+problem%3f%26quot%3b+OR+%26quot%3bfoot+complication%3f%26quot%3b))+OR+(SU+diabetic+foot))+AND+(TI+guideline))&cli0=DT1&clv0=201001-201612&lang=zh-cn&type=1&site=ehost-live

| Titles | Year | Excluded after reading titles | Excluded after reading full texts |
| --- | --- | --- | --- |
| The new NICE guidance on the diabetic foot: a summary from members of the guideline development group | 2015 | Excluded. Not evidence-based guidelines. |  |
| 2012 Infectious Diseases Society of America Clinical Practice Guideline for the diagnosis and treatment of diabetic foot infections. | 2013 |  | Included. |
| Society for Vascular Surgery Releases Guideline on Managing the Diabetic Foot. | 2016 | Not guidelines. |  |
| An exploration of nurses' perceptions regarding the implementation of a best practice guideline on the assessment and management of foot ulcers for people with diabetes. | 2011 | Not guidelines. |  |
| Guideline recommends intensifying treatment of diabetic foot ulcers | 2016 | Not guidelines. |  |
| New NICE guideline on diabetic foot problems published | 2011 | Not guidelines. |  |
| Evaluation of TCOM/HBOT practice guideline for the treatment of foot burns occurring in diabetic patients. | 2015 | Not guidelines. |  |
| Is scintigraphy a guideline method in determining amputation levels in diabetic foot? | 2014 | Not guidelines. |  |
| Implementing the SIGN guideline on the management of diabetes. | 2010 | Not guidelines. |  |

Flow chart:


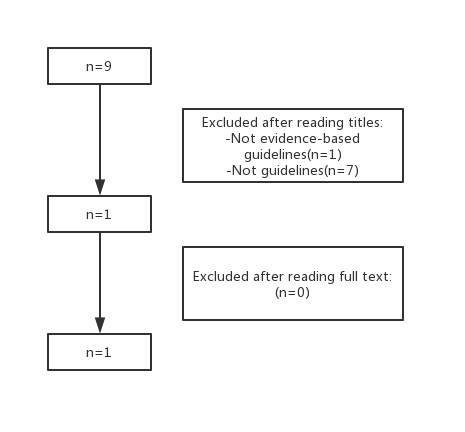


1. Best practice(n=20):

Search strategy: “Diabetic foot AND guidelines”

| Titles |  | Excluded after reading titles | Excluded after reading full texts |
| --- | --- | --- | --- |
| 1. [Prevention and management of diabetic foot](http://www.nhm.gov.in/nrhm-instate/520-standard-treatment-guidelines.html) | 2016 |  | Excluded. Not evidence-based guideline |
| 2. [IDF clinical practice recommendations on the diabetic foot](https://www.idf.org/e-library/guidelines.html)  Published by: International Diabetes Federation | 2017 |  | Excluded. Not evidence-based guideline |
| 3. [Standards of medical care in diabetes - 2018](http://professional.diabetes.org/content/clinical-practice-recommendations)  Published by: American Diabetes Association | 2018 | Excluded. Not evidence-based guideline |  |
| 4.[Best practice recommendations for the prevention and management of diabetic foot ulcers](https://www.woundscanada.ca/health-care-professional/education-health-care-professional/advanced-education/12-healthcare-professional/110-supplements)  Wounds Canada | 2017 |  | Excluded. Not evidence-based guideline |
| 5. href="#Skin%20&%20Soft%20Tissue" Clinical practice guideline for the diagnosis and treatment of diabetic foot infections  Infectious Disease Society of America | 2012 |  | Included. |
| 6. [Prevention and management of diabetic foot](http://www.nhm.gov.in/nrhm-instate/520-standard-treatment-guidelines.html)  Ministry of Health and Family Welfare, Government of India | 2016 | Excluded. Duplication with number1. |  |
| 7. href="https://www.nice.org.uk/guidance/ng19" Diabetic foot problems: prevention and management  National Institute for Health and Care Excellence | 2016 |  | Included. |
| 8. href="http://iwgdf.org/guidelines/" IWGDF guidance on the prevention and management of foot problems in diabetes  International Working Group on the Diabetic Foot | 2015 |  | Included. |
| 9.[IWGDF guidance on footwear and offloading interventions to prevent and heal foot ulcers in patients with diabetes](http://iwgdf.org/guidelines/)  International Working Group on the Diabetic Foot | 2015 |  | Included. |
| 10. [IWGDF guidance on use of interventions to enhance the healing of chronic ulcers of the foot in diabetes](http://iwgdf.org/guidelines/)  International Working Group on the Diabetic Foot | 2015 |  | Included. |
| 11. [IWGDF guidance on the diagnosis and management of foot infections in persons with diabetes](http://iwgdf.org/guidelines/)  International Working Group on the Diabetic Foot | 2015 |  | Included. |
| 12. href="http://iwgdf.org/guidelines/" IWGDF guidance on the diagnosis, prognosis and management of peripheral artery disease in patients with foot ulcers in diabetes  International Working Group on the Diabetic Foot | 2015 |  | Included. |
| 13. [Standards of medical care in diabetes - 2018](http://professional.diabetes.org/content/clinical-practice-recommendations)  American Diabetes Association | 2018 | Excluded. Duplication with number3. |  |
| 14. [Microvascular complications and foot care](http://care.diabetesjournals.org/content/41/Supplement_1/)  American Diabetes Association | 2018 |  | Excluded. Not evidence-based guideline. |
| 15. [Best practice recommendations for the prevention and management of diabetic foot ulcers](https://www.woundscanada.ca/health-care-professional/education-health-care-professional/advanced-education/12-healthcare-professional/110-supplements)  Wounds Canada | 2017 | Excluded. Duplication with number4 |  |
| 16. href="https://vascular.org/research-quality/clinical-practice-documents/clinical-practice-guidelines" The management of diabetic foot  Society for Vascular Surgery; American Podiatric Medical Association; Society for Vascular Medicine | 2016 |  | Included. |
| 17. [Inpatient management of diabetic foot disorders: a clinical guide](http://care.diabetesjournals.org/content/36/9/2862)  American Diabetes Association | 2013 |  | Excluded. Not evidence-based guideline. |
| 18. [Management of patients with peripheral artery disease](http://my.americanheart.org/professional/GuidelinesStatements/UCM_316885_Guidelines-Statements.jsp)  American College of Cardiology Foundation; American Heart Association | 2013 |  | Excluded. Not related |
| 19. [Clinical practice guidelines: foot care](http://guidelines.diabetes.ca/fullguidelines)  Canadian Diabetes Association | 2013 |  | Excluded. Not evidence-based guideline. |
| 20. [Clinical practice guideline for the diagnosis and treatment of diabetic foot infections](#Skin%20&%20Soft%20Tissue)  Infectious Disease Society of America | 2012 | Excluded. Duplication with number5. |  |

Flow chart:


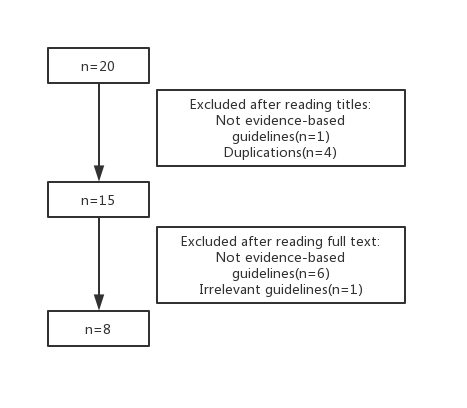


1. CNKI(n=42)，Wangfang Data(38)，VIP data(27)

Search strategy: 糖尿病足[主题] and 指南[篇名] 2010.1-2018.6

Original database:

Articles remains after deleting the duplications:

Flow chart:


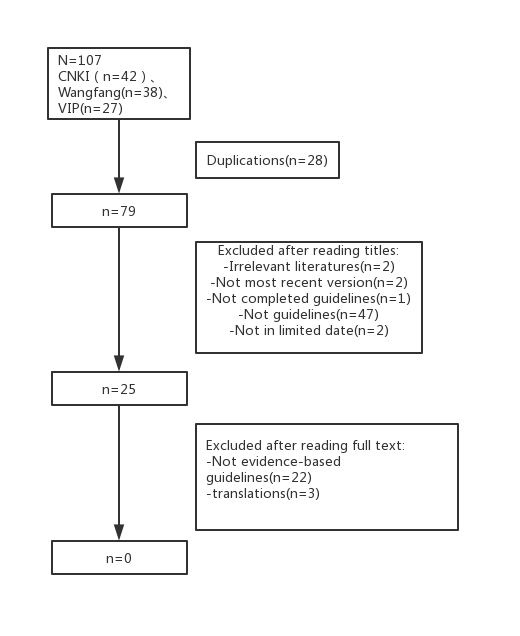


1. Medlive(n=45):

Key words: 糖尿病足

| Titles | Year | Excluded after reading titles | Excluded after reading full text. |
| --- | --- | --- | --- |
| 1. 糖尿病足创面修复专家共识 | 2018 | Not evidence-based guideline. |  |
| 1. [2016 日本糖尿病指南](http://www.medlive.cn/redirect.php?resource=guide_relatedguide&index=1&url=http%3A%2F%2Fguide.medlive.cn%2Fguideline%2F15275) | 2018 |  | Only a very small part of content about DF. |
| 1. 2017《中国糖尿病足诊治指南》解读 | 2017 | Not guideline. |  |
| 1. 糖尿病足介入综合诊治临床指南（第4版） | 2018 |  | Not evidence-based guideline. |
| 1. 2018 澳大利亚糖尿病足指南：糖尿病患者鞋类准备 | 2018 |  |  |
| 1. 2013 澳大利糖尿病患者鞋类准备指南 | 2013 | Not most recent version. |  |
| 1. 2016 共识文件：优化下肢慢性创面愈合中的技术应用 | 2016 | Not guideline. |  |
| 1. 2011 ESVS指南：严重肢体缺血和糖尿病足的管理 | 2011 |  | Only a small part of diabetic foot. |
| 1. 2017 波兰糖尿病患者管理指南 | 2017 |  | Only a small part of diabetic foot. |
| 1. 2017 IDF临床实践建议：糖尿病足 | 2017 | Not guideline |  |
| 1. 中国糖尿病足诊治指南 | 2017 |  | Not evidence-based guideline. |
| 1. 2016 BOA联合建议：糖尿病足多学科治疗 | 2016 | Not guideline. |  |
| 1. 《2016 APMA/SVS/SVM临床实践指南：糖尿病足的管理》 关于糖尿病足溃疡预防的解读 | 2016 | Not guideline. |  |
| 1. 《2016 APMA/SVS/SVM临床实践指南：糖尿病足的管理》（摘译） | 2016 | Translation. |  |
| 1. 国际糖尿病足工作组关于鞋袜和减压的指南推荐要点 | 2015 | Translation. |  |
| 1. 《国际糖尿病足工作组糖尿病足的预防指南》 要点选译 | 2015 | Translation. |  |
| 1. 《国际糖尿病足工作组糖尿病足慢性创面处理指南》中文译文 | 2015 | Translation. |  |
| 1. SVS-2016临床指南： 糖尿病足管理 | 2016 | Translation. |  |
| 1. 2016 APMA/SVS/SVM临床实践指南：糖尿病足的管理 | 2016 |  | Included. |
| 1. 2015 WHS指南：糖尿病足溃疡的治疗（更新版） | 2015 |  | Included. |
| 1. 糖尿病足溃疡中医循证临床实践指南 | 2015 |  | Not evidence-based guideline. |
| 1. 2012 澳大利亚糖尿病足网指南：糖尿病足溃疡的管理 | 2012 |  | Not most recent version. |
| 1. 2015 NICE指南：糖尿病足问题的预防和管理 | 2015 |  | Included. |
| 1. 《国际糖尿病足工作组关于糖尿病足感染的诊断与处理指南》解读 | 2015 | Not guideline. |  |
| 1. 2015 UHMS临床实践指南：使用高压氧疗法治疗糖尿病足溃疡 | 2015 |  | Included. |
| 1. 2015 IWGDF指南：糖尿病患者足部感染的诊断和管理 | 2015 |  | Included. |
| 1. 2015 IWGDF指南：糖尿病足溃疡患者外周动脉疾病的诊断，预后和管理 | 2015 |  | Included. |
| 1. 2015 IWGDF指南：高危糖尿病患者足溃疡的预防 | 2015 |  | Included. |
| 1. 2015 IWGDF指南：减荷以及穿鞋指导预防和治疗糖尿病患者足溃疡 | 2015 |  | Included. |
| 1. 2015 IWGDF指南：应用干预措施提高慢性糖尿病足溃疡的愈合 | 2015 |  | Included. |
| 1. 2014 APMA共识指南：通过优化减荷管理糖尿病足溃疡 | 2014 |  | Not evidence-based guideline |
| 1. 2014 美国糖尿病足溃疡管理指南 | 2014 |  | Not evidence-based guideline. |
| 1. 2012 ADS澳大利亚糖尿病足网：糖尿病足溃疡的管理（临床更新） | 2012 |  | Not most recent version. |
| 1. 2013 糖尿病足诊治指南 | 2013 |  | Not evidence-based. |
| 1. 2013 住院患者糖尿病足管理指南 | 2013 |  | Not evidence-based guideline. |
| 1. 2012 自体干细胞移植规范化治疗下肢慢性缺血性疾病的专家共识 | 2012 | Not guideline. |  |
| 1. 2013 2型糖尿病患者合并下肢动脉病变的筛查与规范管理 | 2013 | Irrelevant literature. |  |
| 1. 2013 IDF 老年2型糖尿病管理全球指南 |  |  | Not evidence-based guideline. |
| 1. 2013 ADS 澳大利亚糖尿病足网：糖尿病患者穿鞋指导指南 | 2013 | Duplication. |  |
| 1. 莫匹罗星软膏预防和治疗创面金黄色葡萄球菌感染的使用建议(2010年) | 2010 | Not guideline. |  |
| 1. 2011 IWGDF 糖尿病足溃疡外周动脉病诊疗指南 | 2011 | Not most recent version. |  |
| 1. 2011 IWGDF 糖尿病足感染治疗实践指南 | 2011 | Not most recent version. |  |
| 1. 2011 IWGDF 糖尿病足溃疡面与溃疡创面床管理指南 | 2011 | Not most recent version. |  |
| 1. 2011 IWGDF 糖尿病足处置和预防实践指南 | 2011 | Not most recent version. |  |
| 1. 2012美国感染病学会(IDSA)临床指南：糖尿病足感染的诊断和治疗 | 2012 |  | Included. |

Flow chart:


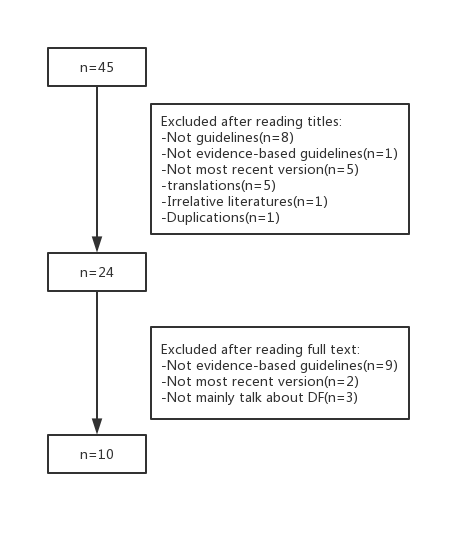

Supplement: S1 Attachment — (DOCX) [file pone.0217555.s001.docx]
